# Minimum Hydration Shells of Small Polar Organic Hydrogen-Bond Acceptors in Explicit Water Clusters

**DOI:** 10.3390/molecules31203583

**Published:** 2026-10-09

**Authors:** Eduardo Romero-Montalvo, Jesús Iván Salazar-Barrientos, José Manuel Guevara-Vela, Samuel García-García, Evelio Francisco, Tomás Rocha-Rinza, Ángel Martín Pendás

**Affiliations:** 1 Instituto de Química, Universidad Nacional Autónoma de México, Circuito Exterior, Ciudad Universitaria, Coyoacán, Mexico City C.P. 04510, Mexico; romero.montalvo.eduardo@gmail.com (E.R.-M.); ji.salazarbarrientos@comunidad.unam.mx (J.I.S.-B.); 2 School of Engineering and Physical Sciences, Heriot-Watt University, Edinburgh EH14 4AS, Scotland, UK; j.guevara-vela@hw.ac.uk; 3 Department of Analytical and Physical Chemistry, University of Oviedo, E-33006 Oviedo, Spain; uo283609@uniovi.es (S.G.-G.); evelio@uniovi.es (E.F.)

**Keywords:** organic molecules, solvation, water clusters, aqueous organic chemistry, solvent effects, interacting quantum atoms

## Abstract

Water plays an active role in organic chemistry, yet the number of explicit water molecules required to describe the local solvation environment of small organic substrates remains poorly established. Herein, we examine the solvation of five small polar hydrogen-bond (HB) acceptors using explicit molecular clusters containing two to twelve water molecules. Conformational searches, electronic structure calculations, and Interacting Quantum Atoms (IQA) energy decomposition were combined to assess energetic convergence and the nature of solute–water interactions. Binding and water deformation energies converge at approximately ten water molecules, defining a practical minimum hydration shell for these substrates. Solute–water interactions are largely confined to the first solvation shell, within ca. 3.5 Å of the heteroatom of the organic substrate, while more distant water molecules recover a bulk-like local environment. Except for trimethylamine, the strongest solute–water contact is weaker than the average HB among water molecules. IQA further shows that differences among interaction energies are governed mainly by the exchange-correlation component, revealing an important covalent contribution to hydrogen bonding. Multiacceptor hydrogen-bonded sites preferentially form water networks around only one HB acceptor rather than bridged independent hydration spheres around each HB acceptor. These results provide practical criteria for constructing compact, chemically meaningful explicit solvation models in aqueous organic chemistry.

## 1. Introduction

Water has become an increasingly important reaction medium in organic chemistry because it can accelerate reactions and modify selectivity with respect to conventional organic solvents [1,2]. Following the seminal work of Rideout and Breslow on the hydrophobic acceleration of Diels–Alder reactions in water [3], Aqueous Organic Chemistry (AOC) has grown into an active field [4,5], driven both by the distinctive behaviour of organic reactions in water and by the low cost, low toxicity, and reusability of this solvent, in line with the principles of green chemistry [6,7]. The broader relevance of aqueous environments in organic chemistry is also evident in biochemical processes [8] and in microscopic water droplets, which can behave as microreactors that accelerate organic transformations [9,10,11,12].

The appeal of AOC is not limited to the replacement of organic solvents with a greener medium. Water can alter reaction pathways by stabilising polar structures, by organising reactants via hydrogen bonding (HB), and by accelerating transformations that are inefficient or inaccessible using other common conditions [4,5]. In some cases, water behaves as a catalyst-like component of the reactive system; in others, it participates directly in proton transfers or in the stabilisation of developing charges along the reaction coordinate [13,14]. The first expression of water’s chemical role is, however, solvation: before water can direct or participate in reactivity, it must organise itself around the organic substrate. For small molecules containing heteroatoms, this local solvation pattern depends on the intrinsic ability of the solute to accept HBs and on the competition between solute–water contacts on one hand and the surrounding water–water HB network on the other. Thus, the solute–water HB interactions in AOC are part of a collective environment whose organisation can influence molecular reactivity in organic chemistry.

This network view has a direct consequence for molecular modelling: the explicit treatment of water molecules becomes necessary whenever the local HB environment is part of the chemical problem under consideration. The water hydrogen-bond network acquires a bulk-like local character only when the number of H_2_O molecules in the system is large enough for H_2_O⋯H_2_O contacts to develop [15,16,17,18]. Nevertheless, microsolvation models commonly rely on a limited number of explicit water molecules, while determining the appropriate number and spatial distribution of these molecules remains a non-trivial problem that is not always addressed through systematic convergence analysis [19,20,21,22,23,24,25]. In other words, the minimum number of explicit water molecules required to describe the hydration of small polar organic solutes remains insufficiently established. The determination of this minimum hydration shell is not only a question of cluster convergence, but also a practical requirement to construct explicit solvation models that are large enough to represent the local water network without introducing unnecessary computational cost.

Given these circumstances, we study the aqueous solvation of five small polar organic HB acceptors, namely acetone, *E*-azomethane, dimethyl ether, methyl acetate, and trimethylamine, which are representative of functional groups commonly encountered in AOC [26]. We examine explicit molecular clusters containing between two and twelve water molecules, generated through conformational searches aided by a genetic algorithm and refined by electronic structure calculations. This cluster series, along with the Interacting Quantum Atoms (IQA) energy partition, allows us to follow how the interaction energies among (i) water monomers and the organic substrate and (ii) surrounding water molecules evolve with cluster size in order to identify the onset of convergence. The IQA real-space energy decomposition permits us to assess whether the organic solute acts as a local or extended perturbation of the water network, to compare the strength of solute–water contacts with that of water–water HBs, and to separate covalent and ionic contributions to the interaction energy among the constituents of the investigated molecular clusters. In this way, we interpreted the minimum hydration shell obtained from the energetic convergence established herein in terms of the spatial range and the nature of the underlying solute–water interactions. Before proceeding further in the paper, we would like to point out a few remarks regarding the scope of our investigation. The electronic structure calculations presented and discussed herein relate to static molecular clusters at 0 K. Hence, our results do not describe the conformational dynamic changes associated with the thermal motion of water. The transferability of our results to room temperature would require finite-temperature molecular dynamics simulations which would provide information on population fluctuations and the interconversion of hydrated structures of small polar molecules which might be engaged in the formation of HBs with H_2_O. Still, this paper addresses the size of the molecular cluster at which the energetic and the interaction descriptors of a solvation model based on electronic structure calculations and wavefunction analysis are insensitive to the addition of further water monomers. In other words, we look at static clusters that represent a minimum hydration shell for the execution of meaningful, yet practical quantum mechanical calculations in molecular clusters related to AOC.

## 2. Theoretical Framework

The Quantum Theory of Atoms in Molecules (QTAIM) provides a real-space partition of a molecular system into atomic basins, and it is based on the topological properties of the electron density, ρ(r). Each atomic basin is bounded by zero-flux surfaces of ∇ρ(r) so that atomic and fragment properties can be obtained by integration over well-defined regions of real space [27]. This partition is especially useful for the examination of intermolecular forces, e.g., HBs, because it allows the interaction between a solute and the surrounding water molecules to be analysed in terms of chemically meaningful atomic or molecular fragments.

The Interacting Quantum Atoms (IQA) approach builds on the QTAIM partition of the 3-D space and decomposes the electronic energy into intra- and inter-atomic contributions [28,29]. For a molecular system divided into QTAIM atoms, the total electronic energy can be written as

(1) $$E=\sum_{A}E_{\mathrm{intra}}^{A}+\frac{1}{2}\sum_{A\neq B}E_{\mathrm{inter}}^{AB}\;,$$

wherein $E_{\mathrm{intra}}^{A}$ is the energy associated with atom *A*, and $E_{\mathrm{inter}}^{AB}$ is the interaction energy between atoms *A* and *B*. The inter-atomic interaction energy can be further split into a classical and an exchange-correlation contribution:

(2) $$E_{\mathrm{inter}}^{AB}=V_{\mathrm{cl}}^{AB}+V_{\mathrm{xc}}^{AB}.$$

Here, the classical term, $V_{\mathrm{cl}}^{AB}$, contains the electrostatic interactions between the nuclei and electron densities of atoms *A* and *B*; hence, $V_{\mathrm{cl}}^{AB}$ is related to ionicity. On the other hand, $V_{\mathrm{xc}}^{AB}$ accounts for exchange-correlation effects and is commonly associated with the covalent component of the interaction between basins *A* and *B*.

In the present work, IQA energies are collected at the molecular-fragment level. Thus, for two molecular fragments or monomers, G and H, the fragment interaction energy is obtained by summing the corresponding atomic pair contributions,

(3) $$E_{\mathrm{inter}}^{\mathrm{GH}}=\sum_{A\in G}\sum_{B\in H}E_{\mathrm{inter}}^{AB}\;,$$

with analogous definitions for $V_{\mathrm{cl}}^{\mathrm{GH}}$ and $V_{\mathrm{xc}}^{\mathrm{GH}}$. This fragment-level description allows us to compare solute–water and water–water interactions on the same rigorous footing.

### Formation Energy and Pairwise Redistribution of Deformation Energies

The formation energy of a cluster G⋯ H⋯ can be written as the difference between the energy of the cluster and those of the isolated fragments:

(4) $$\Delta E_{\mathrm{form}}=E^{G\cdots H\cdots }-\sum_{G}E_{\mathrm{iso}}^{G}.$$

Within the IQA framework, this quantity can be expressed as the sum of fragment deformation energies and pairwise fragment interaction energies,

(5) $$\Delta E_{\mathrm{form}}=\sum_{G}E_{\mathrm{def}}^{G}+\sum_{G\neq H}E_{\mathrm{inter}}^{\mathrm{GH}},$$

wherein

(6) $$E_{\mathrm{def}}^{G}=E_{\mathrm{net}}^{G}-E_{\mathrm{iso}}^{G}.$$

Here, $E_{\mathrm{net}}^{G}$ is the energy of fragment G within the cluster G⋯H⋯ and $E_{\mathrm{iso}}^{G}$ is the energy of the same fragment in its isolated equilibrium geometry. The deformation energy therefore measures the energetic cost associated with distorting a fragment from its isolated minimum-energy geometry to the configuration that it adopts within the cluster. This change in the nuclear configuration results in a change in the corresponding electronic density. Therefore, the deformation energy also entails an energetic cost due to the alteration of electron density of a monomer with respect to the charge distribution of this molecule in its isolated equilibrium geometry.

It is useful to express $\Delta E_{\mathrm{form}}$ as a sum of effective pairwise interaction energies. To this end, the deformation energy of each fragment is redistributed among its pairwise contacts in proportion to the corresponding IQA interaction energies. The effective interaction between fragments G and H is then defined as

(7) $$E_{\mathrm{inter}}^{\;\prime \;\mathrm{GH}}=E_{\mathrm{inter}}^{\mathrm{GH}}+\frac{E_{\mathrm{inter}}^{\mathrm{GH}}}{\sum_{J\neq G}E_{\mathrm{inter}}^{\mathrm{JG}}}E_{\mathrm{def}}^{G}+\frac{E_{\mathrm{inter}}^{\mathrm{GH}}}{\sum_{J\neq H}E_{\mathrm{inter}}^{\mathrm{JH}}}E_{\mathrm{def}}^{H}.$$

With this definition, the cluster formation energy can be written as

(8) $$\Delta E_{\mathrm{form}}=\sum_{G<H}E_{\mathrm{inter}}^{\;\prime \;\mathrm{GH}}.$$

Sauza de la Vega et al. [30] proved the validity of expression (8). The quantities $E_{\mathrm{inter}}^{\;\prime \;\mathrm{GH}}$ therefore allow us to interpret $\Delta E_{\mathrm{form}}$ as a sum of pairwise interaction energies among the interacting molecules that include the direct IQA interaction energy between two fragments and a corresponding share of the deformation energy required to form the cluster.

The same redistribution can be applied to the classical and exchange-correlation components of the interaction energy between groups G and H. Thus, the effective pairwise interaction energy is decomposed as

(9) $$E_{\mathrm{inter}}^{\;\prime \;\mathrm{GH}}=V_{\mathrm{cl}}^{\;\prime \;\mathrm{GH}}+V_{\mathrm{xc}}^{\;\prime \;\mathrm{GH}},$$

where

(10) $$V_{\mathrm{xc}}^{\;\prime \;\mathrm{GH}}=V_{\mathrm{xc}}^{\mathrm{GH}}\left(1+\frac{E_{\mathrm{def}}^{G}}{\sum_{I\neq G}E_{\mathrm{inter}}^{\mathrm{IG}}}+\frac{E_{\mathrm{def}}^{H}}{\sum_{I\neq H}E_{\mathrm{inter}}^{\mathrm{IH}}}\right),$$

and

(11) $$V_{\mathrm{cl}}^{\;\prime \;\mathrm{GH}}=V_{\mathrm{cl}}^{\mathrm{GH}}\left(1+\frac{E_{\mathrm{def}}^{G}}{\sum_{I\neq G}E_{\mathrm{inter}}^{\mathrm{IG}}}+\frac{E_{\mathrm{def}}^{H}}{\sum_{I\neq G}E_{\mathrm{inter}}^{\mathrm{IH}}}\right).$$

This partition allows the effective solute–water and water–water interactions to be compared not only in magnitude, but also in terms of their classical and exchange-correlation contributions, which are related to ionicity and covalency respectively. In the following sections, $E_{\mathrm{int}}^{\prime \;\mathrm{GH}}$, $V_{\mathrm{cl}}^{\prime \;\mathrm{GH}}$, and $V_{\mathrm{xc}}^{\prime \;\mathrm{GH}}$ are used to assess the spatial range, relative strength, and nature of the interactions that define the local hydration environment of the small organic substrates addressed in this investigation.

## 3. Computational Details

Conformational searches were carried out for the OS⋯(H_2_O)_n_ clusters, where OS denotes the organic substrate and n= 2–12. The OSs considered were CH_3_COCH_3_, *E*−CH_3_N=NCH_3_ (where *E* indicates the *E* isomer in the *E*/*Z* naming of the IUPAC), CH_3_OCH_3_, CH_3_COOCH_3_, and N(CH_3_)_3_. We took into account only organic substrates which act as hydrogen-bond acceptors at localised heteroatoms. HB donors, molecules containing both HB donor and acceptor sites, and especially zwitterionic species can generate qualitatively different hydration patterns because they introduce additional directional interactions or even, in the last-mentioned case, strong dominant charge–dipole contributions. We considered progesterone as an illustrative HB multiacceptor system. For each cluster size and organic substrate, between 12 and 20 initial candidate structures were generated from random configurations. These structures were optimised with the PBE/def2-SVP approximation [31,32], and the resulting stationary points were confirmed as bona fide local minima by harmonic frequency calculations. Additional low-energy candidates were generated with a genetic algorithm as implemented in the Cluster v1.3 program [33]. For each system, the genetic algorithm was run for up to 30 generations, and structures not already present in the corresponding population were added to the pool of candidate minima. Given the rapid growth of the configurational space with cluster size, the genetic algorithm was used to obtain a robust sampling of the low-energy region rather than an exhaustive enumeration of all possible minima. The robustness of the conclusions reached in this way is supported by the fact that the main energetic trends are reproduced across all five small organic solutes addressed and, for the multiacceptor HB considered herein, i.e., progesterone, the dominant hydration-topology preference persists when the analysis is expanded from 3 to 5 and 10 kcal mol^−1^ above the energetic minimum (vide infra). The resulting configurations were then reoptimised at the MP2/cc-pVDZ level of theory [34], using the RI [35,36] and RIJCOSX [37] approximations together with the cc-pVDZ/C [38] and def2/J [39] auxiliary basis sets. All electronic structure calculations were performed with the program Orca 5 [40]. The IQA energy partitions were carried out for the selected MP2/cc-pVDZ minima using electron densities computed with the M06-2X/def2-TZVP methodology [28,29,41]. The IQA integrations were performed with the AimAll v19.10.12 program [42]. Because the numerical integration of IQA energies can be sensitive to the quality of the underlying density and integration grid [43], particularly for large hydrogen-bonded clusters, enlarged integration grids were used to reduce numerical errors in the atomic basin integrations. We point out that the computational protocol discussed above was intentionally multilevel. We chose every level according to the requirements of a particular stage of the calculation. The Density Functional Approximation (DFA) PBE/def2-SVP provides a computationally efficient method for exploring the large number of structures required during the conformational search. We subsequently refined the resulting low-energy candidates with the RI-MP2/cc-pVDZ approximation, which introduces an explicit treatment of electron correlation at a computational cost that remains practical for the complete set of clusters considered here. Finally, we employed the M06-2X/def2-TZVP method to obtain the electron densities used in the IQA analysis. The M06-2X functional has been extensively applied to the study of non-covalent interactions, while the larger triple-zeta basis provides a more appropriate description of the density for the subsequent real-space partitioning. In connection with the last-mentioned point, Jiménez-Grávalos et al. [44] examined the performance of DFT methods in the IQA energy partition of small water clusters and they found that the principal interaction trends were reproduced with several DFAs when compared with correlated wave-function references. In short, the different approximations of electronic structure theory mentioned above serve the distinct purposes of configurational exploration, structural refinement, and electron-density analysis. To further verify that the conclusions of the present work are not sensitive to the exploited approximation of electronic structure theory, we recalculated the IQA interaction components for a representative subset of the systems using PBE0/def2-TZVP densities on the same MP2/cc-pVDZ geometries and compared them with the corresponding M06-2X/def2-TZVP results (Figures S1 and S2 of the Supporting Information).

## 4. Results and Discussion

A central question in explicit hydration models in AOC is how many water molecules are needed to describe the local environment around an organic substrate to get reliable results without the involvement of unnecessary computational cost. We therefore begin by analysing how the energetic descriptors of the solute–water aggregates change as the number of water molecules increases. Two complementary quantities are considered: the binding energy per monomer, which measures the average stabilisation of the complete aggregate, and the deformation energy of the water molecules, which probes the structural reorganisation of the system required to form the cluster. Together, these quantities allow us to identify the onset of energetic and structural convergence. Once this minimum hydration shell has been established, we use effective IQA interaction energies, $E_{\mathrm{int}}^{\prime \;\mathrm{OS}\cdots H_{2}O}$, to analyse the spatial range, relative strength, and nature of the OS···H_2_O interactions responsible for the binding energies, which depend on the cluster size of the investigated molecular complexes.

### 4.1. Convergence of Binding and Deformation Energies

First, we examined the association of each organic substrate with water clusters via the binding energy per monomer, defined as

(12) $$E_{b}\left(\mathrm{OS}\cdots {\left(H_{2}O\right)}_{n}\right)=\frac{nE\left(H_{2}O\right)+E\left(\mathrm{OS}\right)-E\left(\mathrm{OS}\cdots {\left(H_{2}O\right)}_{n}\right)}{n+1}.$$

**Figure 1:**
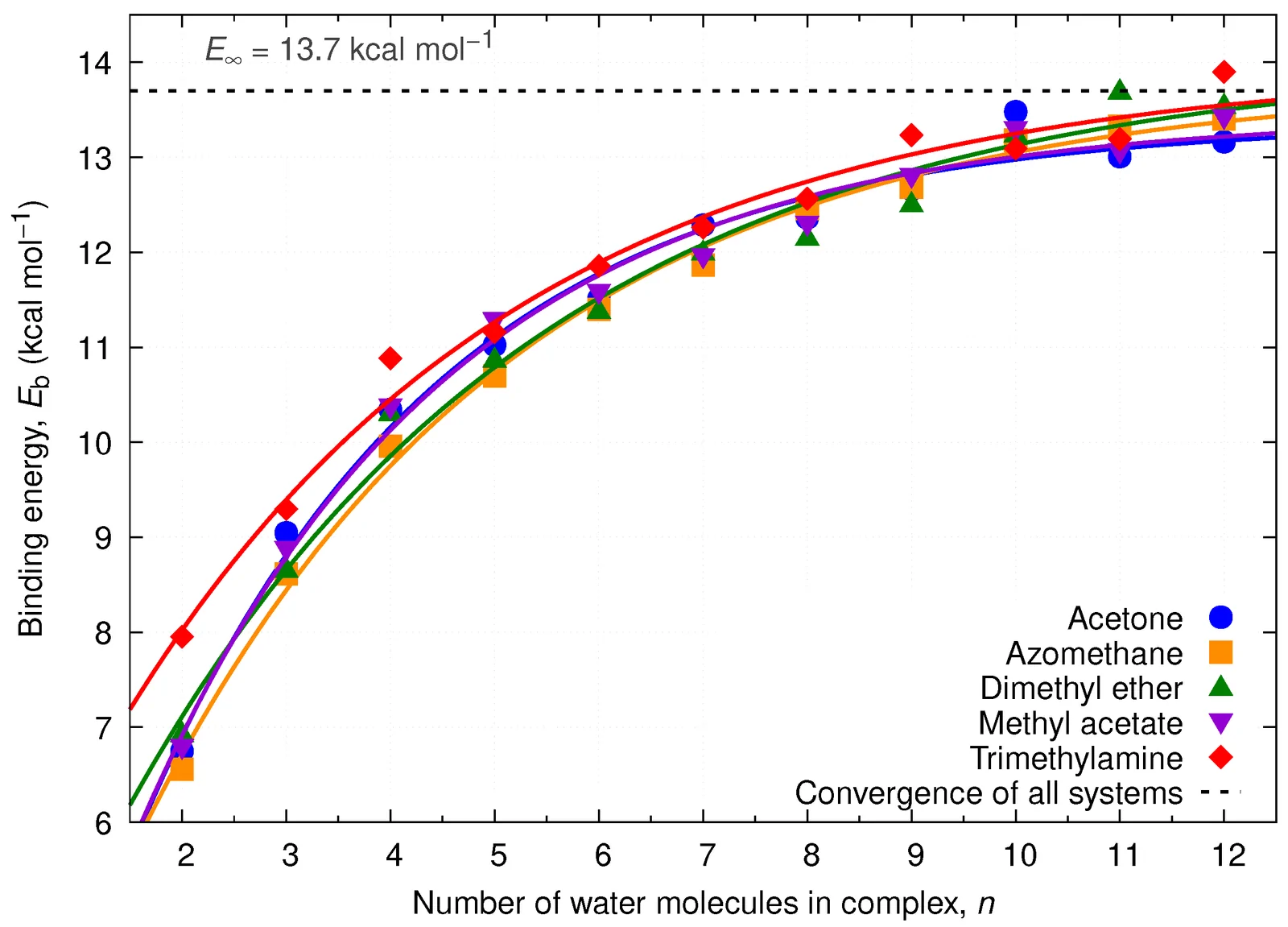
Binding energy per monomer, *E*(OS···(H_2_O)_n_), for each organic solute with water clusters containing between two and twelve water molecules. The data correspond to the single configurations corresponding to the energetic minimum of the addressed OS···(H_2_O)_n_ clusters. Solid lines correspond to per-solvent molecule exponential fits, $E\left(n\right)=E_{\infty }-Ae^{-n/\tau }$, and the dashed line marks the extrapolated asymptotic value, $E_{\infty }=13.7$ kcal mol^−1^.

Here, E(OS), $E(H_{2}O)$, and $E(OS\cdots (H_{2}O)n)$ are the electronic energies of the isolated organic substrate, an isolated water molecule, and the corresponding solute–water cluster, respectively. The denominator n+1 corresponds to the total number of molecular units in the aggregate and therefore converts the total association energy into a size-normalised measure of cluster stabilisation. This normalisation allows clusters of different sizes to be compared directly without assuming a fixed number of intermolecular contacts, since both the number and topology of the solute–water and water–water interactions change as the cluster grows. Figure 1 shows the evolution of $E_{b}(OS\cdots (H_{2}O)n)$ as the number of water molecules in the cluster increases. All five substrates display the same general behaviour; i.e., $E_{b}$ first increases rapidly as water molecules are incorporated into the system and then approaches a common plateau at larger cluster sizes. This convergence indicates that the dominant energetic stabilisation associated with the solute–water aggregate is already captured within a relatively small hydration shell. Although trimethylamine presents slightly larger binding energies for the smallest clusters, consistent with the stronger hydrogen-bond-accepting ability of an sp^3^ atom with regard to the other heteroatoms (vide infra), the differences among the substrates become progressively smaller as the water network grows. This behaviour indicates that, after the first few water molecules have established direct solute–water contacts, the energetic evolution of the clusters is increasingly governed by the development of the H_2_O⋯H_2_O hydrogen-bond network. For all systems, the binding energy approaches an asymptotic value close to 14 kcal mol^−1^, and the fitted curves show that the onset of saturation occurs at about ten water molecules. Thus, from the standpoint of average cluster stabilisation, the addition of water molecules beyond this size produces only minor changes in the energetic description of the hydrated organic substrate.

In order to further test the conclusion that the binding energy approaches its asymptotic regime at approximately ten water molecules, we performed two complementary analyses. First, we examined the stability of the exponential binding-energy fit presented in Figure 1 as more water molecules are included in the system (Table S1). We fitted the same shared-asymptote model using only data up to n=10, n=11, and n=12 in the molecular clusters OS···(H_2_O)_n_. The resulting $E_{\infty }$ values are 13.5, 13.6, and 13.7 kcal mol^−1^, respectively, corresponding to a change of only 1.5% between the n≤10 and n≤12 fits. More importantly, the cluster size at which the value of the fitted binding energy first lay within 5% of $E_{\infty }$ remained essentially unchanged, with a median value of n=10 in all three cases. Second, we performed a hold-out test in which we excluded the binding energies calculated explicitly for n=11 and n=12 from the fit (Figure S3). A model fitted using only the n≤10 data predicts the binding energies for the largest clusters with a mean absolute error of 0.2 kcal mol^−1^ and a mean absolute percentage error of 1.8%. Additionally, the explicitly calculated binding energies over the n= 10–12 interval span only 0.5 kcal mol^−1^ on average, corresponding to 3.4% of the n=12 binding energy. These results show that the onset of binding-energy convergence at approximately ten water molecules is already encoded in the calculated n≤10 data and does not depend on the inclusion of the final two cluster sizes.

We point out that it is also possible to compute $E_{\infty }$ in Figure 1 by plotting $E_{b}\left(\mathrm{OS}\cdots {\left(H_{2}O\right)}_{n}\right)$ as a function of $\frac{1}{n}$ instead of n. We obtained linear relationships shown in the left part of Figure S4 with $R^{2}=0.964$, which is slightly slower than that of Figure 1 ($R^{2}=0.977$). The extrapolated value is in this case $E_{\infty }=14.5\;\mathrm{kcal}/\mathrm{mol}$, which is in good agreement with $E_{\infty }=13.7\;\mathrm{kcal}/\mathrm{mol}$ reported in Figure 1 (the corresponding error is 5.5%). One problem, however, of such linear plots is that the extrapolated value depends very strongly of the values of $E_{b}\left(\mathrm{OS}\cdots \left(H_{2}O_{n}\right)\right)$ for the smallest clusters, n = 2–4, and they have a smaller dependence on the values for the largest clusters. We also note that when n = 10 water molecules, we recover more than 90% of the extrapolated binding energy, in agreement with the above discussion.

**Figure 2:**
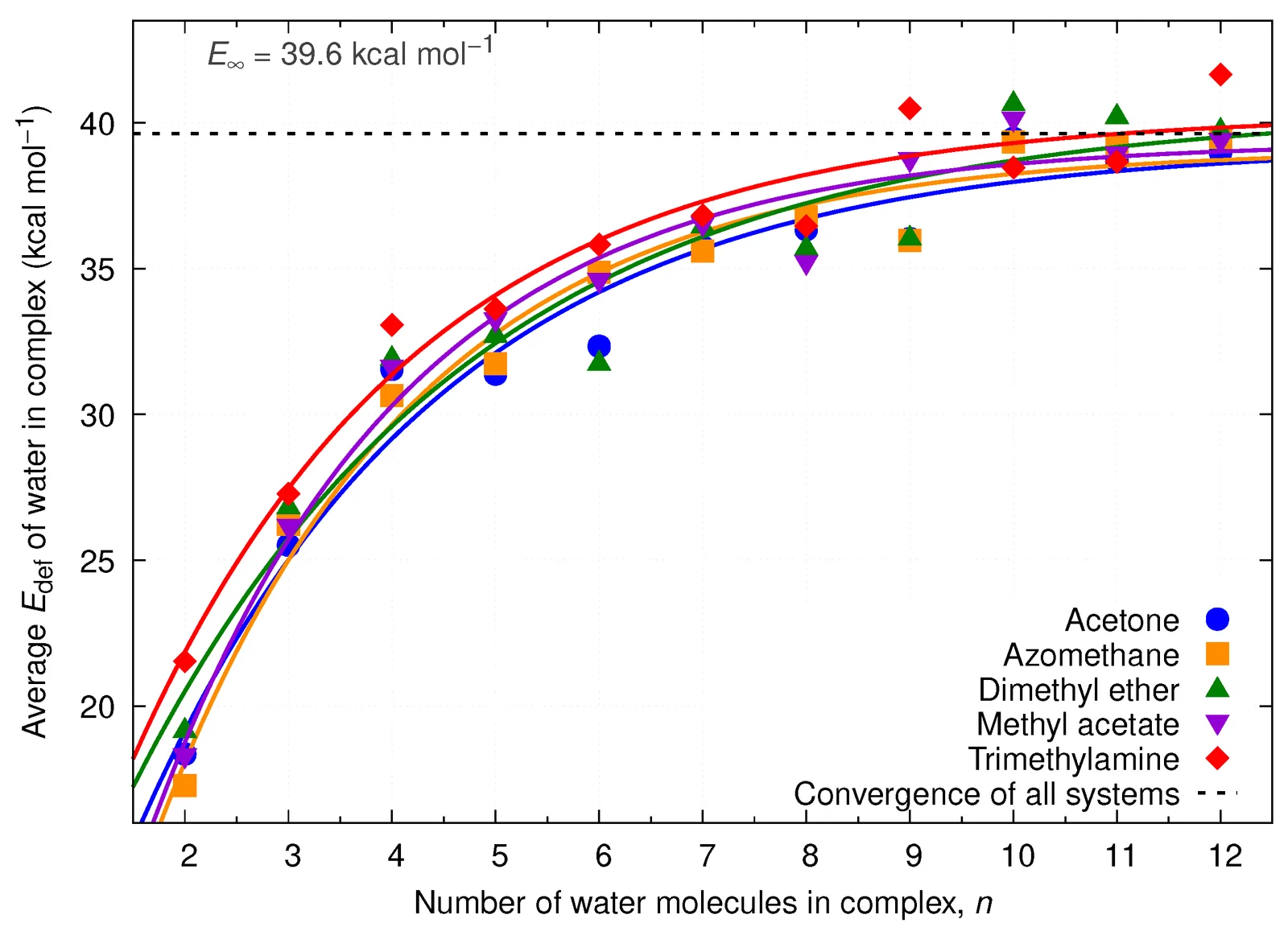
Average deformation energy per water molecule, $\bar{E_{\mathrm{def}}^{H_{2}O}}$, as a function of the number of water molecules in each OS⋯(H_2_O)_n_ (n= 2–12) cluster. The data correspond to the single configurations corresponding to the energetic minimum of the addressed OS···(H_2_O)_n_ clusters. Solid lines correspond to per-solvent-molecule exponential fits, and the dashed line marks the extrapolated asymptotic value, $E_{\infty }=39.6$ kcal mol^−1^.

The saturation of $E_{b}$(OS···(H_2_O)_n_) provides a first indication that the energetic stabilisation of the solute–water aggregate reaches a limiting regime at large cluster sizes. This descriptor is, however, an average quantity: by construction, $E_{b}$(OS···(H_2_O)_n_) is normalised by the total number of monomers in the aggregate. Its plateau therefore reports the convergence of the mean stabilisation of the complete cluster, rather than the behaviour of any individual solute–water or water–water contact. Even with this limitation, the vanishing of the slope of the fitted curves in Figure 1 is informative, because it shows that the energetic change associated with increasing the cluster size becomes progressively smaller. To test whether this energetic saturation is accompanied by the maturation of the hydrogen-bonded water network as H_2_O molecules are incorporated into the system, we next examine the deformation energy of the interacting water monomers. The deformation energy provides a complementary and more direct criterion for the evolution of the HB grid among H_2_O monomers. In this context, $E_{\mathrm{def}}$ measures the energetic cost of distorting each water molecule from its isolated equilibrium geometry to the configuration that it adopts within the solute–water molecular cluster. Unlike $E_{b}$(OS···(H_2_O)_n_), which is an average stabilisation over the complete aggregate, $E_{\mathrm{def}}$ probes the structural reorganisation of the water monomers within the clusters. Figure 2 shows that the average deformation energy increases rapidly for the smallest clusters and then approaches a common asymptotic value around 40 kcal mol^−1^. In the right part of Figure S4, we plotted $E_{\mathrm{def}}^{H_{2}O}$ as a function of $\frac{1}{n}$ and obtain $E_{\infty }=43.3\;\mathrm{kcal}/\mathrm{mol}$, in good agreement with the value indicated in Figure 2. Although the values of $E_{\mathrm{def}}^{H_{2}O}$ might seem considerably high at first sight, Guevara-Vela et al. [16,17] reported that the order of magnitude of this quantity in small water clusters is around tens of kcal/mol in concordance with the results presented herein. The behaviour of $E_{\mathrm{def}}^{H_{2}O}$ indicates that, after the first water molecules have organised around the solute, the addition of further H_2_O monomers no longer produces a substantial change in the internal distortion of the lattice of H-bonded H_2_O molecules. The convergence of $E_{\mathrm{def}}^{H_{2}O}$ occurs slightly earlier than that of $E_{b}$(OS···(H_2_O)_n_), i.e., around nine water molecules, suggesting that the geometric maturation of the water network precedes the complete energetic saturation of the molecular aggregate. Taken together, Figure 1 and Figure 2 show that both the average stabilisation of the cluster and the structural response of the H-bonded water monomer grid reach an asymptotic regime at approximately nine to ten water molecules.

The convergence of both descriptors points to a compact minimum hydration shell for the small polar organic substrates considered herein. In energetic terms, adding water molecules beyond approximately nine to ten molecules produces only minor changes in the average stabilisation of the cluster surrounding a single HB acceptor site. In structural terms, $E_{\mathrm{def}}^{H_{2}O}$ shows that the water network has already reached a mature local arrangement within the same size range. Now, we investigate why such a limited number of water molecules is sufficient to solvate the addressed OS. One possibility is that the organic solute perturbs only nearby H_2_O monomers, while more distant water molecules behave primarily as part of the original H-bonded lattice of H_2_O molecules. To test this idea, we next analyse the spatial decay of the IQA-derived effective interaction energy, $E_{\mathrm{inter}}^{\;\prime \;\mathrm{OS}\cdots H_{2}O}$, between the organic substrate and each water molecule.

### 4.2. Spatial Range of the Solute–Water Interaction

**Figure 3:**
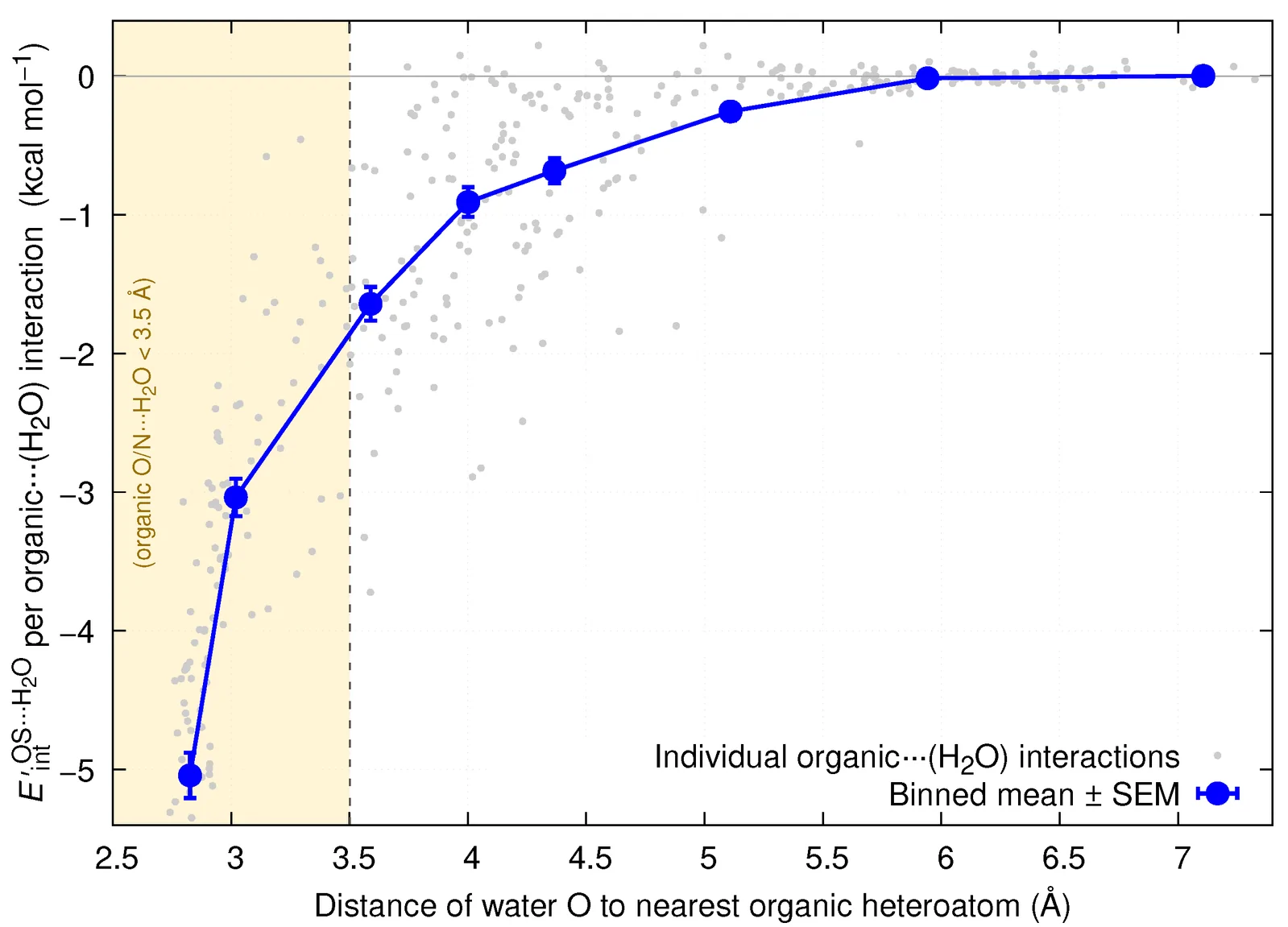
Organic–water effective interaction energy, $E_{\mathrm{int}}^{\;\prime \;\mathrm{OS}\cdots H_{2}O}$, as a function of the distance from each water oxygen to the nearest solute heteroatom. Grey points correspond to individual solute–water interactions, and blue squares show the population-binned mean ± SEM using eight equal-population bins. The shaded region marks the first solvation shell, defined here as distances below 3.5 Å.

Figure 3 shows the effective interaction energy, $E_{\mathrm{inter}}^{\;\prime \;\mathrm{OS}\cdots H_{2}O}$, between the organic substrate and each water molecule as a function of the distance from the H_2_O oxygen to the nearest solute heteroatom. The 3.5 Å boundary used in Figure 3 provides an operational definition of the first solvation shell. This value corresponds to the distance component of the widely used Luzar–Chandler geometrical criterion for hydrogen bonding in liquid water, in which an O⋯O separation below 3.5 Å together with an angular criterion are used to define an individual HB [45,46]. The 3.5 Å distance is also consistent with the first-shell region of the oxygen–oxygen radial distribution function of liquid water [47]. We therefore use 3.5 Å as a practical radial cutoff for the contacts between the oxygen atoms in water molecular and heteroatoms in the organic substrates considered here. This choice is further supported by the present data: the strongest contacts occur predominantly between 2.7 and 3.0 Å, while by 3.5 Å the average of $E_{\mathrm{inter}}^{\;\prime \;\mathrm{OS}\cdots H_{2}O}$ has already decreased substantially. Indeed, the magnitude of the solute–water interaction is strongly determined by short-distance contacts, with the strongest stabilising interactions occurring within the first solvation shell, whereas the magnitude of $E_{\mathrm{inter}}^{\;\prime \;\mathrm{OS}\cdots H_{2}O}$ decays rapidly as water molecules move away from the heteroatom of the OS. Around 3.5 Å, the average interaction has already weakened substantially, and beyond this region the $\mathrm{OS}\cdots H_{2}O$ interaction becomes comparatively unimportant. This extreme distance dependence indicates that the organic substrate acts as a very local perturbation of the network of H-bonded water molecules. Therefore, the convergence observed for the binding and deformation energies can be rationalised in terms of the limited spatial range of the OS⋯H_2_O interaction. Once this first hydration shell is formed, additional water molecules contribute primarily to the surrounding grid of H_2_O⋯H_2_O interactions rather than to stabilising contacts with the organic substrate.

The rapid decay of $E_{\mathrm{inter}}^{\;\prime \;\mathrm{OS}\cdots H_{2}O}$ shows that the convergence of $E_{b}$(OS⋯(H_2_O)_n_) and $E_{\mathrm{def}}^{H_{2}O}$ is more than numerical saturation with cluster size, and it instead reflects a change in the dominant stabilising interactions within the system as more water molecules are incorporated into the molecular cluster. The first solvation shell contains the direct solute–water contacts, whereas more distant water molecules are stabilised primarily by the $H_{2}O$ hydrogen-bonded network. Consequently, once this local shell has formed, further cluster growth has only a minor effect on the average stabilisation of the aggregate and on the deformation pattern of the water molecules. The hydration shell therefore reaches an asymptotic regime once the nearest water molecules around the heteroatom have been established. Beyond spatial locality, it is interesting to compare the strongest $\mathrm{OS}\cdots H_{2}O$ with the characteristic $H_{2}O\cdots H_{2}O$ interactions present in the same clusters, as discussed below.

### 4.3. Competition Between Solute–Water and Water–Water Interactions

**Figure 4:**
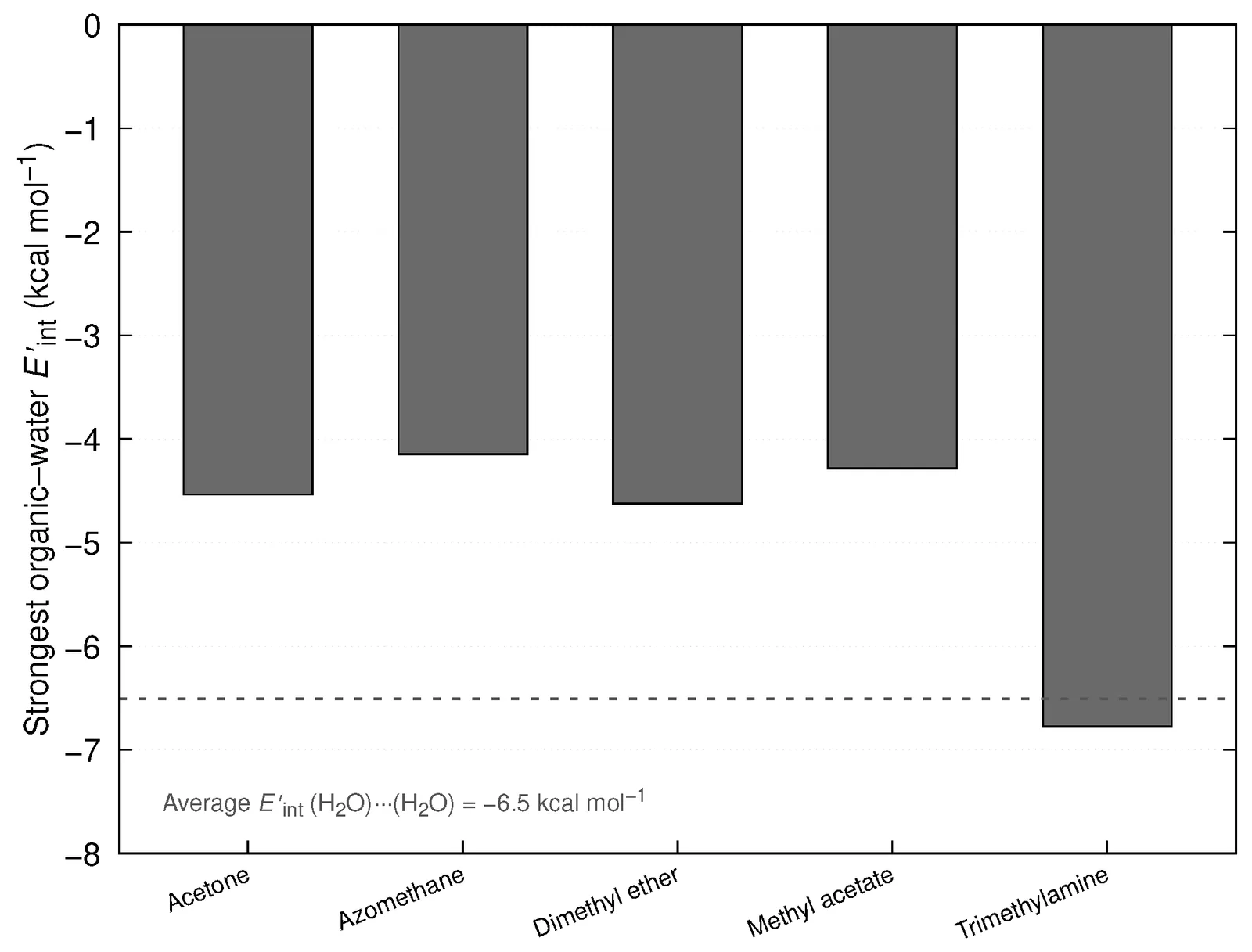
Strongest $\mathrm{OS}\cdots H_{2}O$ effective interaction energy, $E_{\mathrm{int}}^{\;\prime \;\mathrm{OS}\cdots H_{2}O}$, for each solute. The dashed line marks the average water–water effective interaction energy in the same cluster series, $E_{\mathrm{int}}^{\;\prime \;H_{2}O\cdots H_{2}O}=-6.5$ kcal mol^−1^.

Figure 4 compares the strongest $\mathrm{OS}\cdots H_{2}O$ effective interaction energy, $E_{\mathrm{int}}^{\;\prime \;\mathrm{OS}\cdots H_{2}O}$, for each solute with the average $H_{2}O\cdots H_{2}O$ interaction energy in the same cluster series. This comparison shows that, for most substrates, even the strongest direct contact with the organic molecule is weaker than a typical $H_{2}O\cdots H_{2}O$ hydrogen bond. In other words, the interactions of acetone, azomethane, dimethyl ether, and methyl acetate therefore do not outcompete $H_{2}O\cdots H_{2}O$. Instead, the organic substrates are accommodated within the H-bonded H_2_O grid as comparatively weak local perturbations. Trimethylamine is the only exception, with a strongest $\mathrm{OS}\cdots H_{2}O$ contact slightly more stabilising than the average $E_{\mathrm{int}}^{\;\prime \;\mathrm{OS}\cdots H_{2}O}$ interaction. This behaviour is consistent with the slightly larger binding energies observed for trimethylamine in the smallest clusters (Figure 1), for which the direct interaction with the amine nitrogen has a greater relative weight than those corresponding to the other addressed organic substrates before the water network becomes fully developed. The contrast with ion–water clusters is instructive: whereas ion–dipole interactions dominate the organisation of the first and even second hydration shells [30], the neutral organic solutes studied here remain much closer in strength to ordinary $H_{2}O\cdots H_{2}O$ hydrogen bonding. Thus, the compact hydration shell inferred from $E_{b}$(OS⋯(H_2_O)_n_) and $E_{\mathrm{def}}^{H_{2}O}$ reflects not only the short spatial range of the solute–water interaction, but also its limited ability, in most cases, to compete with the hydrogen-bonded H_2_O network.

### 4.4. Classical and Exchange-Correlation Components of the Solute–Water Interaction

**Figure 5:**
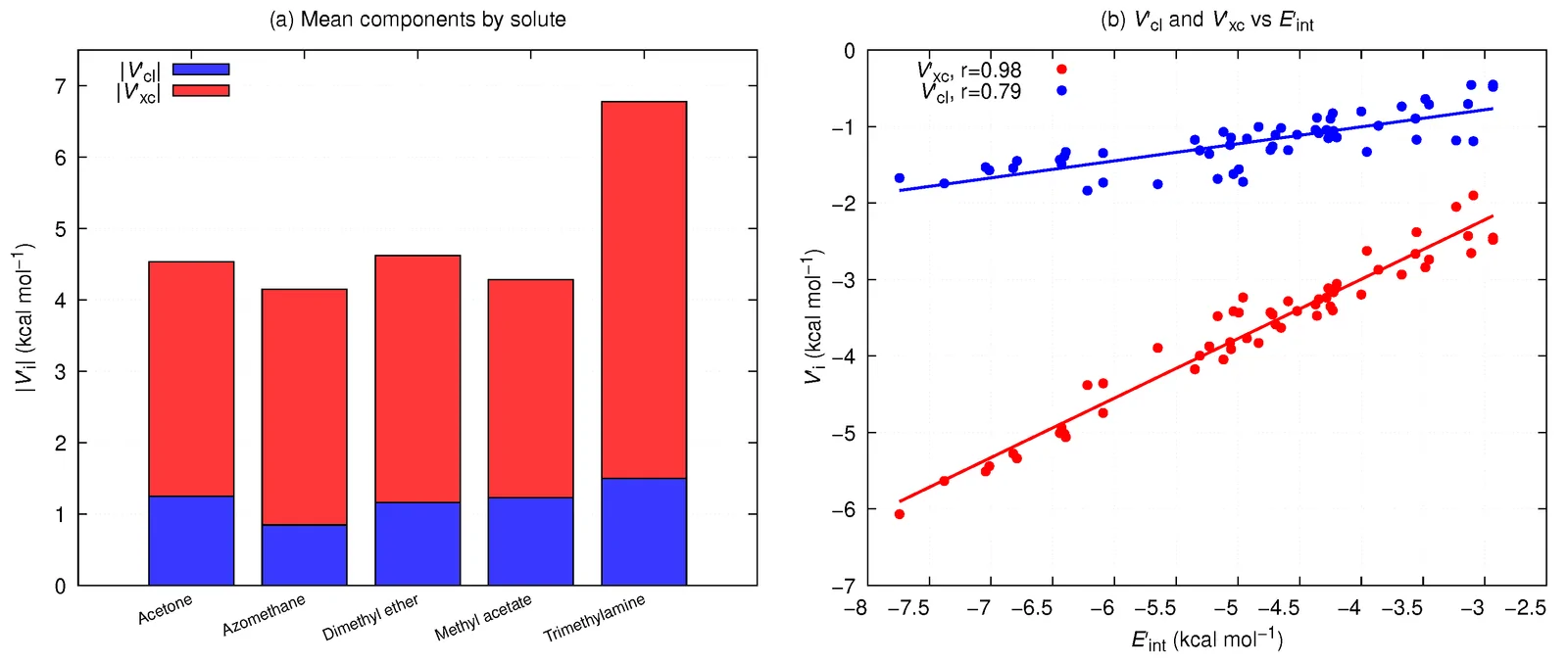
Classical and exchange-correlation components of the $\mathrm{OS}\cdots H_{2}O$ effective interaction energy. (**a**) Mean classical, $V_{\mathrm{cl}}^{\;\prime \;\mathrm{OS}\cdots H_{2}O}$, and exchange-correlation, $V_{\mathrm{xc}}^{\;\prime \;\mathrm{OS}\cdots H_{2}O}$, components for each solute. (**b**) Linear regressions between $V_{\mathrm{cl}}^{\;\prime \;\mathrm{OS}\cdots H_{2}O}$ and $V_{\mathrm{xc}}^{\;\prime \;\mathrm{OS}\cdots H_{2}O}$ on one hand, and the total effective interaction energy, $E_{\mathrm{int}}^{\;\prime \;\mathrm{OS}\cdots H_{2}O}$, on the other.

The previous analysis shows that the $\mathrm{OS}\cdots H_{2}O$ interaction is local and, for most substrates, weaker than the average $H_{2}O\cdots H_{2}O$ HB. A remaining question is whether the differences among the organic solutes arise mainly from classical electrostatic effects or from exchange-correlation contributions associated with electron sharing. This distinction is useful because a purely electrostatic picture would imply that the interaction strength is governed mostly by the polarity of the solute–water contact, whereas a strong dependence on $V_{\mathrm{xc}}^{\prime \;\mathrm{OS}\cdots H_{2}O}$ would indicate a relevant contribution from covalency in the interaction. Figure 5a shows that the exchange-correlation component is the dominant contribution to the $\mathrm{OS}\cdots H_{2}O$ effective interaction energy for all substrates. Although it may seem surprising at first, covalency plays an important role in hydrogen bonding as reported by Martín Pendás and coworkers [48]: “The total interfragment interaction energy is significantly greater than the binding energy, and the exchange-correlation term is usually much larger than the electrostatic interaction; so absolutely speaking, covalent contributions are more important than electrostatic terms. $V_{xc}$ is almost completely composed of the exchange contributions of the H⋯B and A⋯B atomic pairs (The HB referred by the authors is A–H⋯B). This three-centre exchange is quite directional, favouring the commonly found linear arrangement around the HB. Moreover, the important stabilising quantum mechanical exchange between the fragments is almost cancelled by their deformation energies. The sum of deformation and exchange correlation, which plays a similar role in IQA as the exchange repulsion of other energy partitions, is very small. The remaining classical electrostatic term is thus very similar to the overall binding energy.” Subsequent IQA studies of water clusters reached the same conclusion, including that of Guevara-Vela et al. [16] and related studies [17]. This picture is also consistent with other energy decomposition approaches; for example, Phipps et al. [49] review the role of charge-transfer contributions as a measure of covalent character in intermolecular interactions. Figure 5b further shows that $V_{\mathrm{xc}}^{\prime \;\mathrm{OS}\cdots H_{2}O}$ is much better correlated to the total interaction energy than $V_{\mathrm{cl}}^{\prime \;\mathrm{OS}\cdots H_{2}O}$, with a stronger linear correlation and a steeper variation across the range of $E_{\mathrm{inter}}^{\prime \;\mathrm{OS}\cdots H_{2}O}$. In contrast, $V_{\mathrm{cl}}^{\prime \;\mathrm{OS}\cdots H_{2}O}$ remains confined to a narrower energetic interval and changes only moderately as the total interaction becomes more stabilising. Thus, the differences in $\mathrm{OS}\cdots H_{2}O$ interaction strength are controlled primarily by the exchange-correlation contribution, with trimethylamine again showing the largest value. This result indicates that the stronger interaction of the amine is a consequence of the larger covalent character of the corresponding HB contact, as captured by the exchange-correlation component of the IQA interaction energy. This behaviour is expected to extend qualitatively to other simple aliphatic amines, which display broadly comparable hydrogen-bond acceptor strengths, although substitution and steric effects can introduce significant quantitative differences [50]. Accordingly, trimethylamine can be considered as representative of strongly basic, relatively unhindered aliphatic amines rather than of amines as a whole.

The molecular picture that emerges from the IQA analysis is that the compact minimum hydration shell arises from the local character and limited strength of the solute–water interaction which is governed mainly by the exchange-correlation component. This circumstance points to the importance of short-range covalency rather than to a purely long-range electrostatic effect. We recall at this point that the $\mathrm{OS}\cdots H_{2}O$ contacts are, in most cases, weaker than the $H_{2}O\cdots H_{2}O$ hydrogen bonds that stabilise the examined clusters. The interaction energies among the OSs and H_2_O molecules do not surpass those among the H_2_O monomers. Instead, the OS presents as a perturbation which disrupts the H-bonded water grid. Additionally, the effective solute–water interaction decays rapidly with distance and is largely confined to the first solvation shell. Once this first shell has formed, additional water molecules contribute mainly to the recovery of a water-like network around it, which explains why both $E_{b}$(OS⋯(H_2_O)_n_) and $E_{\mathrm{def}}^{H_{2}O}$ reach their asymptotic regimes with a relatively small number of H_2_O molecules.

### 4.5. Hydration of Molecules with Multiple Interaction Sites

The systems considered above contain a single dominant HB-accepting site, and therefore they provide a relatively simple picture of the development of the local hydration environment. Many organic molecules of chemical interest, however, contain several sites that can interact simultaneously with water. To examine how the conclusions obtained for the simpler substrates extend to this situation, we considered progesterone as a representative hydrogen-bond multiacceptor system. Progesterone contains two carbonyl oxygen atoms that can independently act as hydrogen-bond acceptors, giving rise to four possible hydration patterns:

- The water network may become anchored predominantly to only one of the two H_2_O sites.
- A single continuous grid may connect both carbonyl groups.
- The water molecules may form a self-associated cluster without directly hydrogen bonding to either carbonyl oxygen.
- Two independent hydration networks may develop around the two HB acceptor sites.

**Figure 6:**
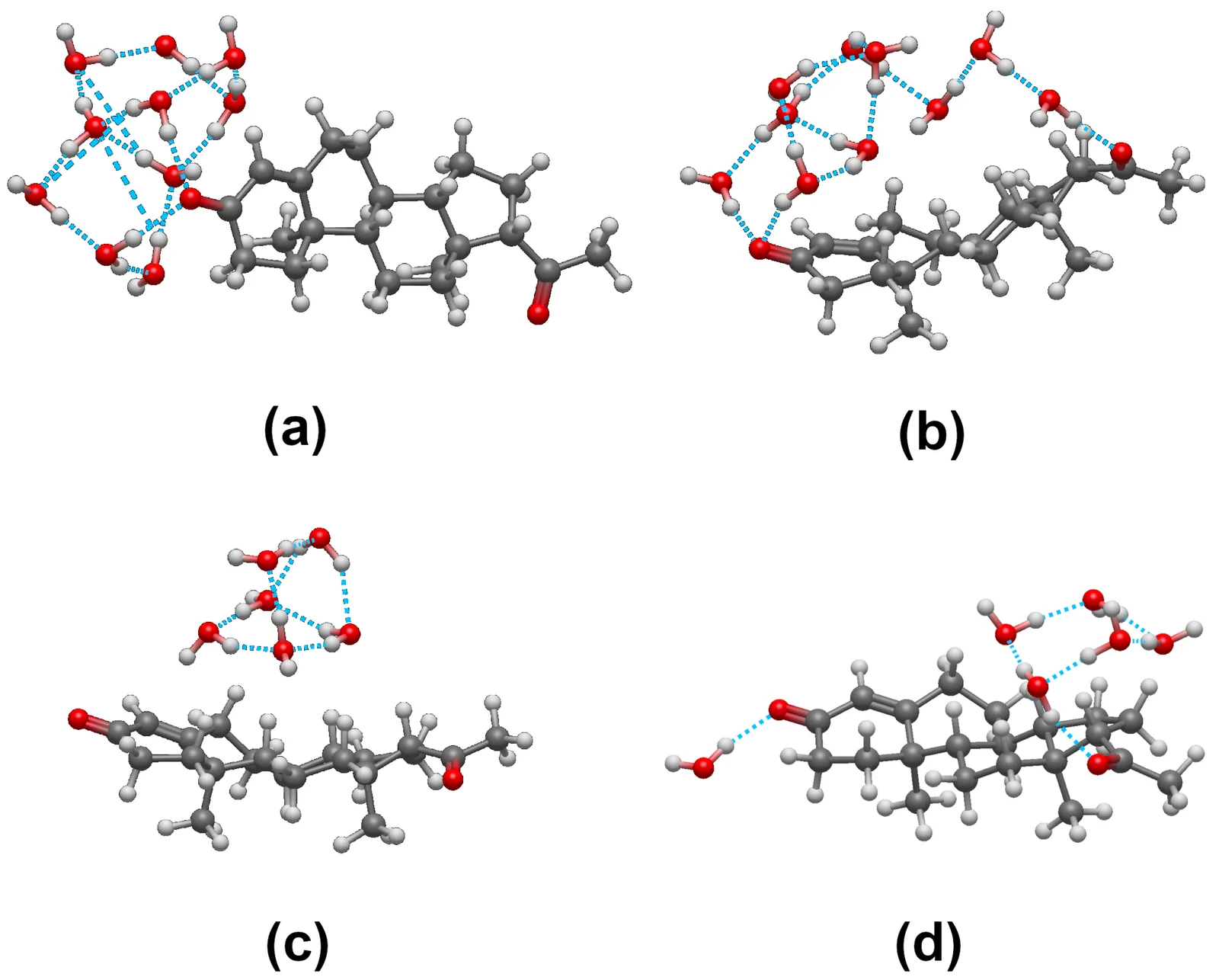
Representative hydration patterns of progesterone within explicit water clusters wherein blue dashed lines indicate hydrogen-bonding interactions. (**a**) A connected water hydrogen-bond network anchored exclusively to one of the two progesterone oxygen atoms. (**b**) A bridged topology in which a single continuous water network connects both oxygen sites. (**c**) A self-associated water cluster that does not form a direct hydrogen bond to either progesterone oxygen atom. (**d**) Two independent hydration structures formed around the two carbonyl oxygen atoms of progesterone. Blue dashed lines indicate hydrogen-bonding interactions.

Representative structures of the first, second, and fourth possibilities are shown in Figure 6. In the most frequently observed pattern, the complete hydrogen-bonded water network is anchored to only one of the two carbonyl oxygen atoms of progesterone (Figure 6a). When both carbonyl oxygen atoms participate in the hydration structure, they can become connected through a single continuous water network, as illustrated in Figure 6b. In a small number of structures, the water cluster remains self-associated without directly hydrogen bonding to either carbonyl oxygen (Figure 6c). The formation of two independent hydration networks around the two carbonyl oxygen atoms was also only rarely observed among the low-energy structures, as discussed below (Figure 6d).

To determine whether these arrangements were characteristic only of the minimum-energy structures or persisted among nearby conformers, we analysed all isomers lying within 3 kcal mol^−1^ of the energetic minimum for each progesterone–water cluster, comprising between two and fourteen water molecules, acting as an organic substrate. The resulting hydration-network topologies are summarised in Table 1. Of the forty-nine low-energy structures considered, forty-two contain a connected water network anchored exclusively to one of the two progesterone oxygen atoms. Only four structures display a bridged topology in which the same water network connects both HB acceptor sites in progesterone, while a single structure contains two independent hydration networks around each carbonyl in progesterone. In the remaining two cases, no water molecule satisfies the geometrical hydrogen-bond criterion with either progesterone oxygen atom. Thus, even when several hydrogen-bond acceptor atoms are available, the water molecules show a marked tendency to remain incorporated into a common hydrogen-bond network anchored to a single site, rather than partitioning into independent solvation spheres around each acceptor.

We repeated the hydration topology analysis using broader windows of 5 and 10 kcal mol^−1^ in order to evaluate whether the conclusions drawn from the original 3 kcal mol^−1^ window depended on the selection of only the lowest-energy structures. The trends with the smallest energetic window prevailed. Single-site-anchored water networks account for 85.7%, 80.7%, and 68.8% of the structures within 3, 5, and 10 kcal mol^−1^ of the corresponding minima, respectively, whereas two independent hydration networks remain uncommon, representing only 2.0%, 3.6%, and 4.5% of the respective ensembles (Table S2). By extending the energy window w.r.t. the energetic minima of the molecular clusters involving progesterone, higher-energy structures represent an increasing fraction of configurations in which the water cluster forms a direct hydrogen bond with neither of the progesterone oxygen atoms. Nevertheless, this circumstance does not alter the conclusion that independent solvation spheres around the two acceptor sites are disfavoured.

This behaviour has a direct implication for the construction of explicit hydration models of organic molecules containing multiple HB acceptors. Increasing the total number of water molecules does not by itself ensure that all chemically relevant sites will acquire an appropriate local hydration environment, because the favourable H_2_O–H_2_O hydrogen-bond network will typically concentrate the available solvent molecules around a single region of the solute. Furthermore, the lowest-energy microsolvated structure is not necessarily the most appropriate to describe a particular chemical process. If a specific functional group or reactive site is the object of the calculation, a somewhat higher-energy isomer in which that site is properly hydrated may provide a more chemically meaningful starting point than the global minimum-energy structure. Consequently, explicit water molecules should be deliberately included around the region of interest, or the conformational sampling should retain low-lying alternative hydration patterns rather than selecting structures solely on the basis of their relative energy. For molecules with multiple HB acceptors, the practical construction of explicit solvation models thus requires consideration not only of the number of water molecules, but also of their spatial distribution and of the chemical process that the model is intended to describe.

## 5. Conclusions

In this work, we examined the aqueous solvation of five small polar organic molecules containing hydrogen-bond acceptors using explicit water clusters and IQA energy decomposition. The convergence of both the binding energy of the molecular cluster and the deformation energy of the water molecules indicates that approximately ten explicit H_2_O monomers are sufficient to reach the asymptotic local hydration regime for the small polar organic hydrogen-bond acceptors considered herein. We stress that neutral HB acceptors or zwitterionic species may require molecular clusters of different sizes and that these systems should be examined separately. The deformation energy shows that the structural maturation of the water network slightly precedes the energetic saturation of the cluster, supporting the use of this size as a practical minimum hydration shell for small polar organic solutes. The IQA analysis provides a molecular interpretation of this convergence. The interaction between the organic solute and the water cluster is strongly spatially localised. The organic molecule mainly perturbs the first solvation shell, within ca. 3.5 Å of the heteroatom, whereas more distant water molecules interact only weakly with the solute and recover a bulk-like local environment. In most cases, the strongest OS···H_2_O contact is weaker than the average H_2_O···H_2_O hydrogen bond, showing that these organic substrates are accommodated as comparatively modest perturbations of the water hydrogen-bond network. Trimethylamine is the main exception, as its amine nitrogen forms a solute–water contact that is competitive with the water–water interaction. The decomposition of the interaction energy further shows that the differences among the solutes arise mainly from the exchange-correlation component, while the classical contribution remains comparatively invariant across the series. Thus, the strength of the solute–water interaction is less strongly governed by a simple electrostatic picture than by the covalent character of the hydrogen-bonding contact, as captured by the exchange-correlation component of the IQA interaction energy. The extension to progesterone further shows that, for molecules containing multiple hydrogen-bond acceptor sites, the number of explicit water molecules alone does not guarantee an appropriate solvation pattern. Low-energy structures preferentially form a connected water network that is often anchored to a single HB acceptor site, indicating that a chemically relevant description may require the consideration of low-lying hydration isomers rather than the global minimum alone. Overall, these results show that small polar organic solutes can be modelled with compact but chemically meaningful explicit hydration shells, and they provide a practical criterion for constructing explicit solvation models in aqueous organic chemistry. Finally, we point out that a direct comparison between the results of our research and the description of the system by means of continuous-medium solvent models (PCM, SMD) or cluster continuous-medium mixed models is not feasible. The PCM and SMD model do not contain individual water molecules and they cannot describe the development of the H_2_O···H_2_O hydrogen-bond network that underlies the convergence analysed in this work. Nevertheless, the results of this investigation are compatible with cluster–continuum approaches in a complementary sense: for the systems studied here, the approximately ten water molecules criterion can provide a physically motivated explicit inner solvation region, while the continuum accounts for the longer-range response of the surrounding solvent in AOC.

## Figures and Tables

**Table 1:** Hydration-network topologies of progesterone⋯H_2_O clusters among isomers lying within 3 kcal mol^−1^ of the minimum-energy structure for each cluster size. “Single-site anchored” denotes a connected water network associated with only one oxygen of progesterone; “Bridged” a single H_2_O network connecting both carbonyl sites in progesterone; “Independent” two independent H_2_O networks around each carbonyl in progesterone; and “No O-site HB” structures without a hydrogen bond with either progesterone oxygen.

n	Low-Energy Isomers	Single-Site Anchored	Bridged	Independent	No O-Site HB
2	7	7	0	0	0
3	5	5	0	0	0
4	5	5	0	0	0
5	7	7	0	0	0
6	6	3	1	1	1
7	5	4	0	0	1
8	2	2	0	0	0
9	2	2	0	0	0
10	3	2	1	0	0
11	2	2	0	0	0
12	2	0	2	0	0
13	2	2	0	0	0
14	1	1	0	0	0
Total	49	42	4	1	2

## Data Availability

The XYZ files containing the structural populations generated and analysed in this study are available in the Supplementary Materials. All other data supporting the findings of this study are contained within the article.

## Supplementary Materials

The following supporting information can be downloaded at https://www.mdpi.com/article/10.3390/molecules31203583/s1.
