# Supplementary material for: Minimum Hydration Shells of Small Polar Organic Hydrogen-Bond Acceptors in Explicit Water Clusters
Source: Molecules. 2026 Oct 9;31(20):3583. doi: 10.3390/molecules31203583 (PMC13707625; doi:10.3390/molecules31203583)
Supplement: Supplementary file 1 [file molecules-31-03583-s001.zip › molecules-4603335-supplementary.pdf]

Minimum Hydration Shells of Small Polar Organic Hydrogen Bond Acceptors in Explicit Water Clusters
Supplementary Information

Eduardo Romero-Montalvo[a], Jesús Iván Salazar-Barrientos[a], José Manuel Guevara-Vela[b], Samuel García-García[c], Evelio Francisco[c], Tomás Rocha-Rinza[a], Ángel Martín Pendás[c,*]

[a] *Instituto de Química, Universidad Nacional Autónoma de México, Circuito Exterior, Ciudad Universitaria, Coyoacán, C.P. 04510, Ciudad de México, Mexico.*
[b] *School of Engineering and Physical Sciences, Heriot-Watt University, Edinburgh EH14 4AS, Scotland, U.K.*
[c] *Department of Analytical and Physical Chemistry, University of Oviedo, E-33006 Oviedo, Spain.*

*Corresponding author: ampendas@uniovi.es

1. Sensitivity of the IQA decomposition to the density-functional level

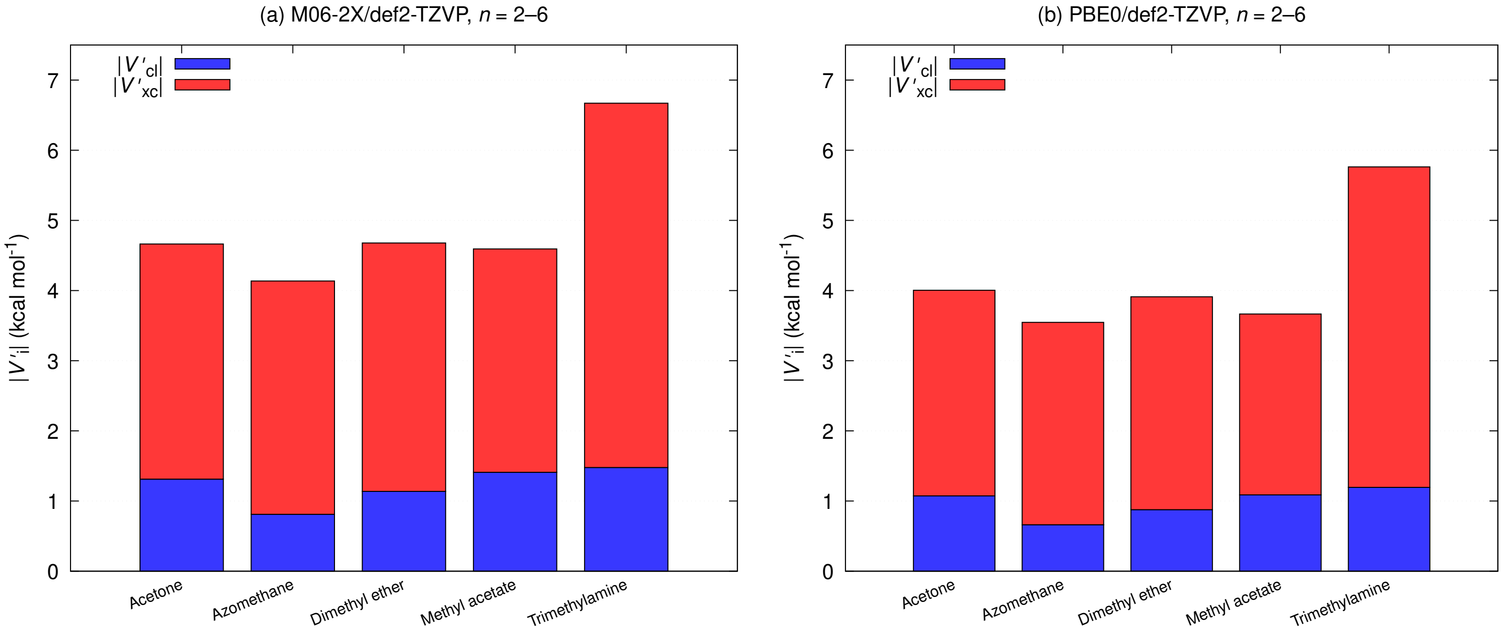

Figure S1: Comparison of the IQA interaction-energy decomposition obtained from M06-2X/def2-TZVP and PBE0/def2-TZVP electron densities for representative n = 2 − 6 clusters, evaluated on the same MP2/cc-pVDZ geometries. The classical, $|V'_{\mathrm{cl}}|$, and exchange-correlation, $|V'_{\mathrm{xc}}|$, contributions retain the same qualitative relationship with both density functionals. In particular, the exchange-correlation contribution remains dominant for all five solutes, and trimethylamine exhibits the largest contribution at both levels of theory.

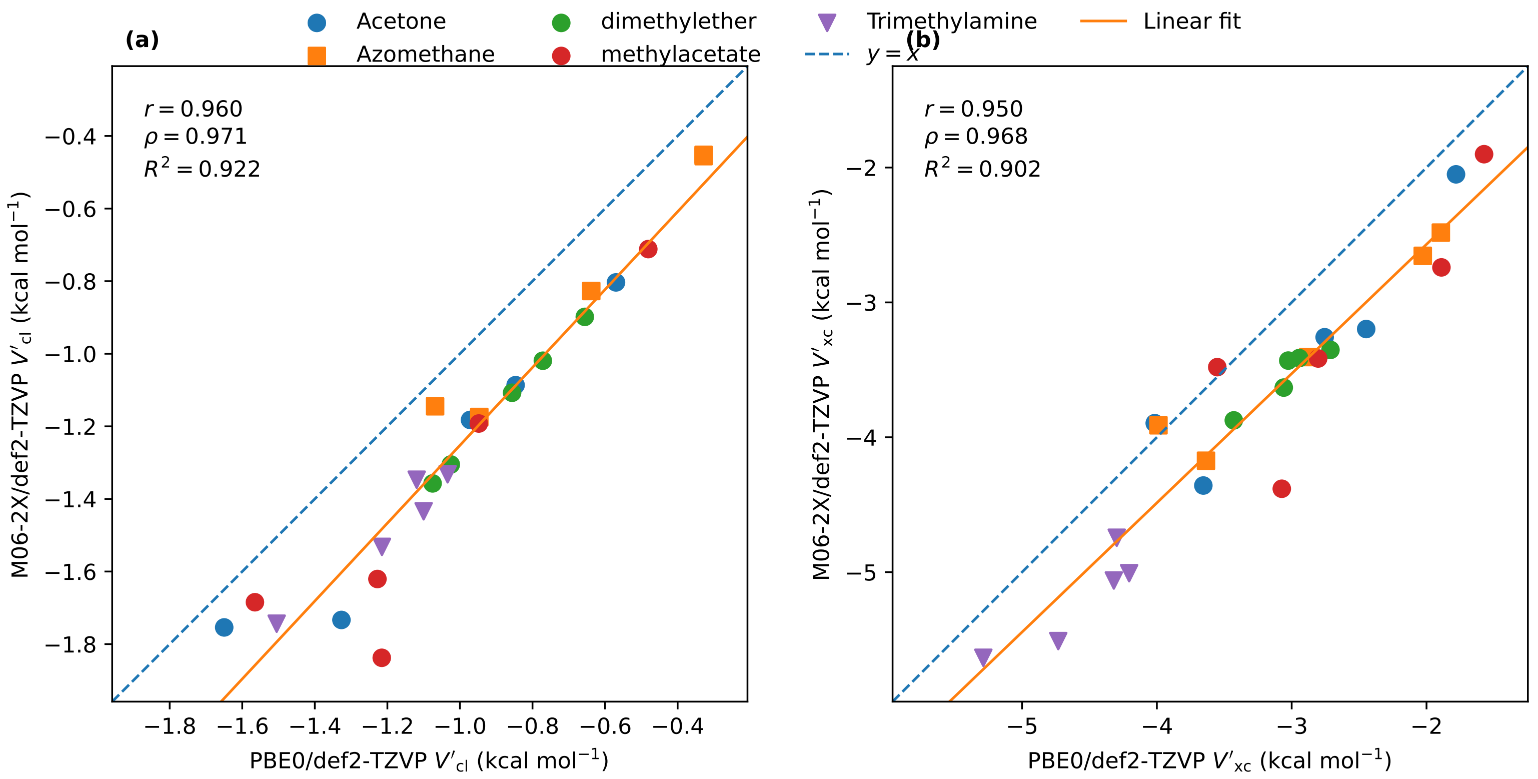

Figure S2: Point-by-point comparison of the IQA interaction-energy components obtained from M06-2X/def2-TZVP and PBE0/def2-TZVP electron densities for the same set of solute–water interactions, evaluated on identical MP2/cc-pVDZ geometries. Panel (a) compares the classical contribution, V'_{cl}, and panel (b) the exchange-correlation contribution, V'_{xc}. The dashed line indicates perfect numerical agreement between the two density-functional levels, while the solid line represents the linear regression. Although M06-2X yields systematically more attractive interaction components than PBE0, the point-by-point correspondence remains strongly linear for both V'_{cl} ($r = 0.960$, $\rho = 0.971$, $R^2 = 0.922$) and V'_{xc} ($r = 0.950$, $\rho = 0.968$, $R^2 = 0.902$). Thus, the choice of functional affects the absolute magnitude of the IQA terms but does not alter the relative trends or the qualitative interpretation of the solute–water interactions.

2. Asymptote fit

Table S1: Stability of the shared-asymptote binding-energy fit with respect to the largest cluster size included in the analysis. The exponential model was refitted using data truncated at $n_{\max} = 10$, 11, and 12 water molecules. E_∞ is the shared asymptotic binding energy obtained from each fit, τ is the corresponding exponential decay parameter, and the 5% convergence range gives the cluster size at which the fitted binding energy first lies within 5% of E_∞. The persistence of the $n \approx 10$ convergence criterion across all three fitting windows shows that the inferred onset of saturation is insensitive to the inclusion of the largest calculated clusters.

$n_{\max}$	E_∞ (kcal mol^{-1})	τ	5% convergence, n
10	13.50	3.15	9–10
11	13.58	3.24	9–10
12	13.70	3.39	10

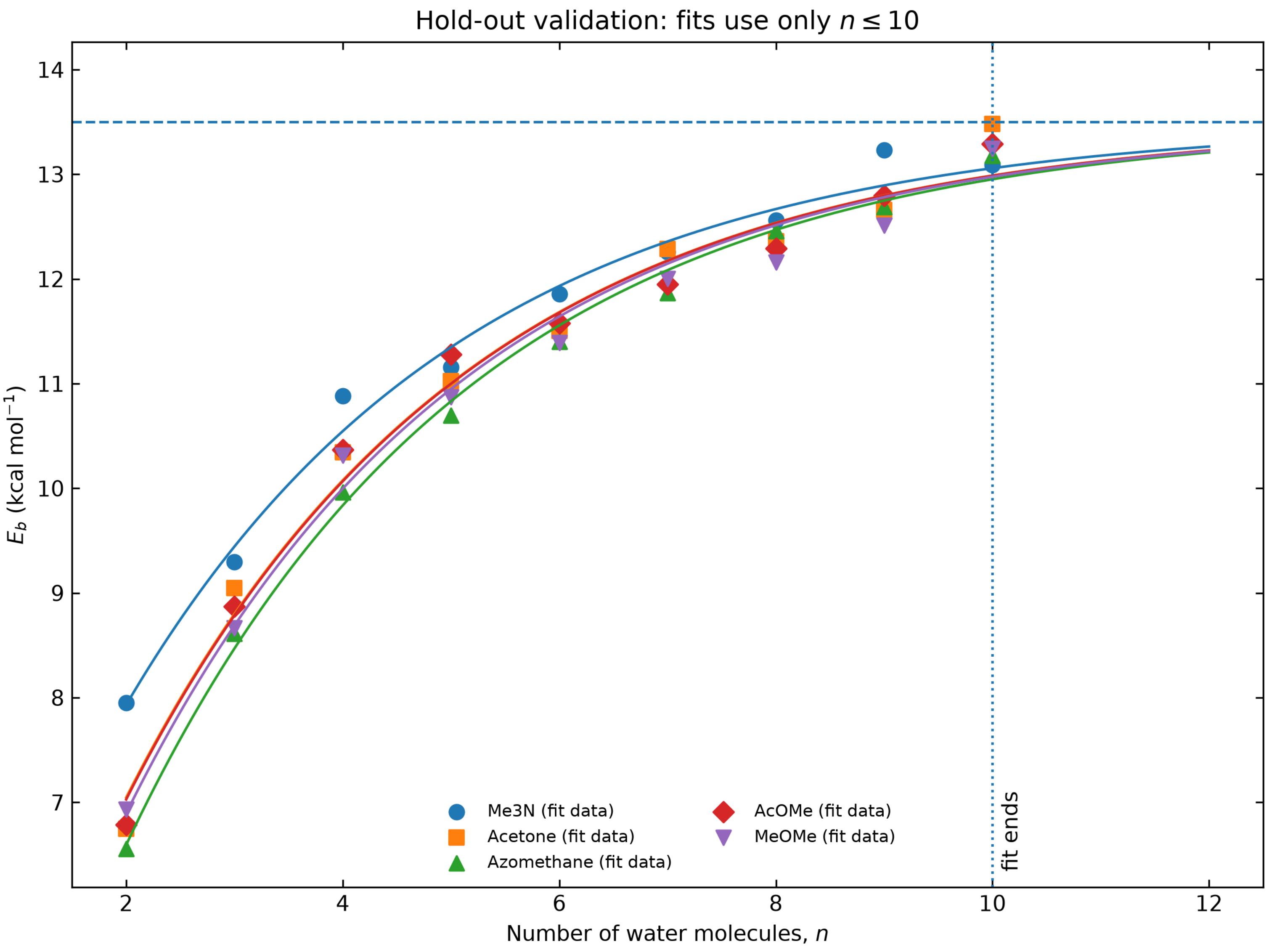

Figure S3: Hold-out validation of the binding-energy convergence analysis. The exponential models were fitted using only the data for clusters with $n \leq 10$ water molecules. The explicitly calculated $n = 11$ and $n = 12$ values, shown as open symbols, were excluded from the fitting procedure and used only as validation points. The good agreement between these hold-out data and the extrapolated curves shows that the convergence behaviour is already determined by the $n \leq 10$ data. The horizontal dashed line marks the shared asymptotic value obtained from the $n \leq 10$ fit, and the vertical dotted line indicates the largest cluster size included in the fit.

3. Relationships of $E_b(\mathrm{OS}\cdots(\mathrm{H_2O})_n)$ and $E_{\mathrm{def}}^{\mathrm{H_2O}}$ as a function of $\frac{1}{n}$

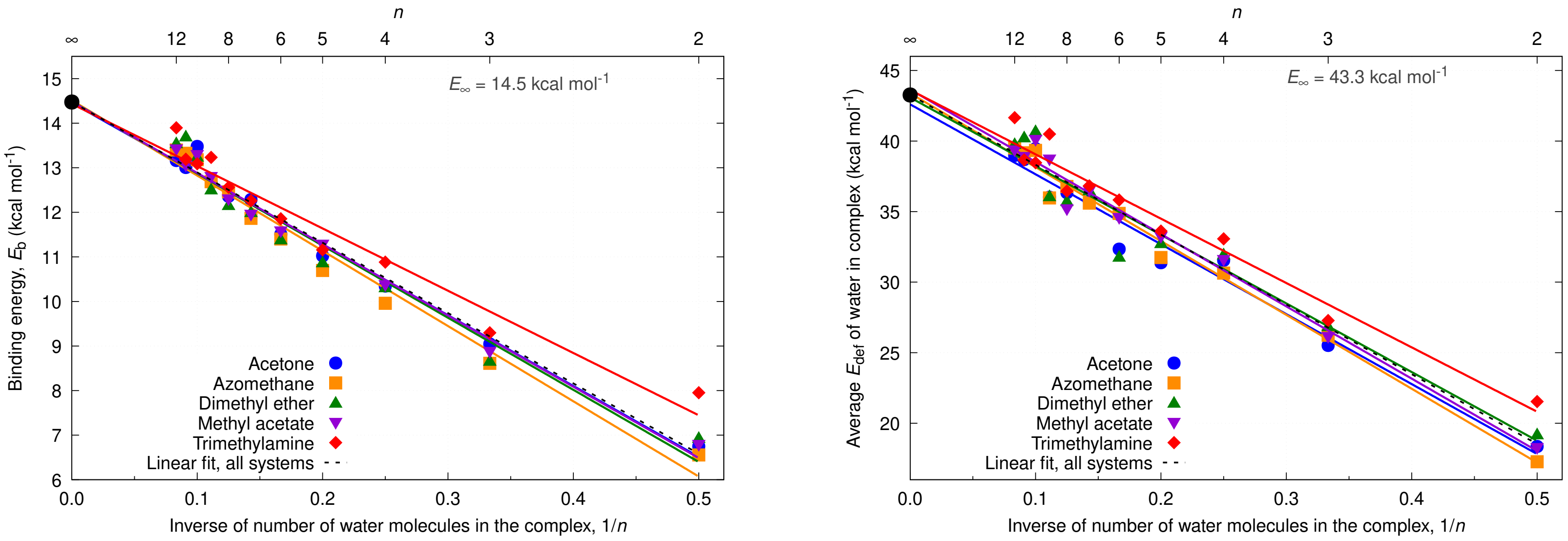

Figure S4: (Left) Binding energy per monomer, $E_b(\mathrm{OS}\cdots(\mathrm{H_2O})_n)$, for each organic solute with water clusters containing between two and twelve water molecules as a function of $\frac{1}{n}$. The computed asymptotic value is 14.5 kcal/mol. (Right) Ditto for $E_{\mathrm{def}}^{\mathrm{H_2O}}$ with an asymptotic value 43.3 kcal/mol.

4. Sensitivity of progesterone hydration topologies to the energy window

Table S2: Dependence of the progesterone hydration-network topology on the energy window used to define the structural ensemble. Percentages are given relative to the number of structures retained within each energy cutoff.

Energy window	Structures	Single-site anchored	Bridged	Independent	No O-site HB
3	49	42 (85.7%)	4 (8.2%)	1 (2.0%)	2 (4.1%)
5	83	67 (80.7%)	4 (4.8%)	3 (3.6%)	9 (10.8%)
10	157	108 (68.8%)	9 (5.7%)	7 (4.5%)	33 (21.0%)
